# Antisense PMO Found in Dystrophic Dog Model Was Effective in Cells from Exon 7-Deleted DMD Patient

**DOI:** 10.1371/journal.pone.0012239

**Published:** 2010-08-18

**Authors:** Takashi Saito, Akinori Nakamura, Yoshitsugu Aoki, Toshifumi Yokota, Takashi Okada, Makiko Osawa, Shin'ichi Takeda

**Affiliations:** 1 Department of Molecular Therapy, National Institute of Neuroscience, National Center of Neurology and Psychiatry, Kodaira, Tokyo, Japan; 2 Department of Pediatrics, School of Medicine, Tokyo Women's Medical University, Shinjuku, Tokyo, Japan; 3 Research Center for Genetic Medicine, Children's National Medical Center, Washington, District of Columbia, United States of America; Hospital Vall d'Hebron, Spain

## Abstract

**Background:**

Antisense oligonucleotide-induced exon skipping is a promising approach for treatment of Duchenne muscular dystrophy (DMD). We have systemically administered an antisense phosphorodiamidate morpholino oligomer (PMO) targeting dystrophin exons 6 and 8 to a dog with canine X-linked muscular dystrophy in Japan (CXMD_J_) lacking exon 7 and achieved recovery of dystrophin in skeletal muscle. To date, however, antisense chemical compounds used in DMD animal models have not been directly applied to a DMD patient having the same type of exon deletion. We recently identified a DMD patient with an exon 7 deletion and tried direct translation of the antisense PMO used in dog models to the DMD patient's cells.

**Methodology/Principal Findings:**

We converted fibroblasts of CXMD_J_ and the DMD patient to myotubes by FACS-aided MyoD transduction. Antisense PMOs targeting identical regions of dog and human dystrophin exons 6 and 8 were designed. These antisense PMOs were mixed and administered as a cocktail to either dog or human cells *in vitro*. In the CXMD_J_ and human DMD cells, we observed a similar efficacy of skipping of exons 6 and 8 and a similar extent of dystrophin protein recovery. The accompanying skipping of exon 9, which did not alter the reading frame, was different between cells of these two species.

**Conclusion/Significance:**

Antisense PMOs, the effectiveness of which has been demonstrated in a dog model, achieved multi-exon skipping of dystrophin gene on the FACS-aided MyoD-transduced fibroblasts from an exon 7-deleted DMD patient, suggesting the feasibility of systemic multi-exon skipping in humans.

## Introduction

Antisense oligonucleotides (AON) have been reported to modulate splicing of pre-mRNA transcribed from mutated genes and to restore a normal reading frame in several diseases. Duchenne muscular dystrophy (DMD), a degenerative muscle disorder caused mainly by nonsense or frame-shift mutations of the dystrophin gene, is one of the diseases that could be treated by AON-mediated exon skipping. Previously reported studies were conducted *in vitro*, in animal models, and as patient intervention studies, and they showed restorations of the reading frame in dystrophin mRNA and recoveries of dystrophin protein expression [1], [2], [3]. Among the several AON chemistries that have been introduced thus far, a phosphorodiamidate morpholino oligomer (PMO) and 2'-O-methyl phosphorothioate (2'OMe) oligomer are promising candidates owing to their stabilities and efficacies, and they are now undergoing phase I-II clinical trials in the United Kingdom and the Netherlands, respectively [4], [5]. The AON-mediated exon skipping is already in a late early stage of clinical application; therefore, it is rational to translate pre-clinical animal model knowledge into a patient-based study.

We have previously reported that the systemic administration of an antisense PMO for canine X-linked muscular dystrophy in Japan (CXMD_J_) achieved restoration of dystrophin and amelioration of symptoms [6]. CXMD_J_ harbors a splice site mutation within the splice acceptor site of intron 6 of the dystrophin gene. The mutation disrupts the splicing of exon 7, and thus the dystrophin mRNA lacks exon 7 [7]. In CXMD_J_, multiple skipping of exons 6 and 8 restores the reading frame, and the multi-exon skipping approach is expected to expand the number of DMD cases potentially treatable by exon skipping [8]. CXMD_J_ is an ideal model of multi-exon skipping, and we hope to translate the results to human patients. However, in the road to ongoing clinical trials, *in vitro* assays on patient cells are indispensable.

To date, antisense sequences used for exon skipping in DMD animal models have not been directly applied to a DMD patient having the same type of exon deletion. We identified an exon 7-deleted patient (referred to as DMD 8772) and tried direct translation of the antisense PMO design from a DMD dog model to the DMD patient. We tried *in vitro* multi-exon skipping with the same antisense PMO that was used in CXMD_J_ in the patient's cells before attempting delivery of the PMO into the patient.

Which cells should be used for *in vitro* dystrophin exon skipping is controversial. Myoblasts are usually employed simply because they express enough dystrophin as mRNA and protein, but collecting them requires an invasive muscle biopsy. In cases where myoblasts were not available, it had been reported that the dystrophin mRNA was detected in lymphocytes and fibroblasts by nested RT-PCR. Some studies actually demonstrated the success of exon skipping in mRNA of lymphoblastoid cells and fibroblasts [9], [10], but the restoration of dystrophin protein could not be analyzed in these cells because their transcripts were illegitimate and too low to be translated into gene products [11]. As another alternative, fibroblasts are converted to myotubes by MyoD transduction [4], [12], [13]. Transduced cells express dystrophin mRNA and protein, but achievement of sufficient protein expression is challenging [14], [15], [16]. In this study, we addressed this issue by introducing a retroviral vector co-expressing MyoD and green fluorescent protein (GFP) and flow cytometry, and then quantified the dystrophin expression of the cells to evaluate the feasibility of exon skipping.

We first report multiple skipping of dystrophin exons 6 and 8 in the DMD patient's cells and translation of the unified antisense PMO design from a DMD dog model to a human based on the MyoD-transduction method utilizing flow cytometry.

## Results

### Mutation analysis of DMD 8772

**Figure 1:**
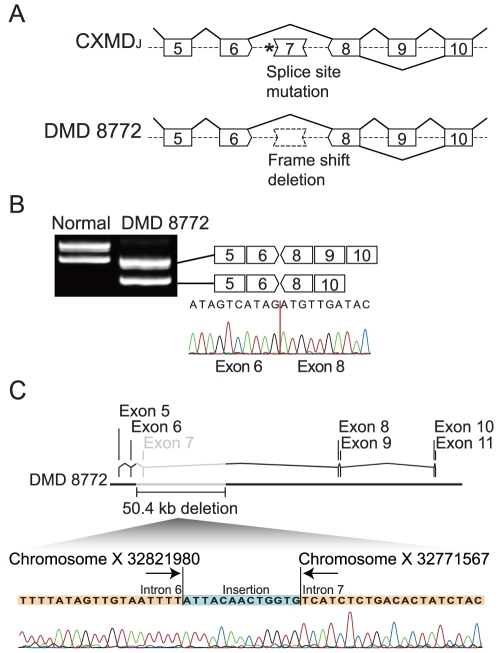
Mutation analysis of DMD 8772. (A) Splice-site mutation of a splice acceptor site in intron 6 (asterisk) excludes exon 7 from dog dystrophin mRNA. Frame-shift deletion of dystrophin exon 7 in DMD 8772 was diagnosed by MLPA analysis. Skipping of exon 9 is a frequent splice variant. Both ends of the schematic box of the exon represent a phase of the codon (see detail, Yokota et al. 2009). (B) RT-PCR and sequence analysis of dystrophin mRNA using normal and DMD 8772 lymphocytes. Double bands due to a splicing variant of exon 9 were observed. (C) Breakpoint analysis of DMD 8772 revealed a 50.4 kb deletion from intron 6 to intron 7, and the insertion of 13 bases of unknown origin.

DMD 8772, a 22-year-old man, manifested severe muscle weakness, wheelchair dependency, and mild cardiac dysfunction. No evidence of dystrophin protein had been observed on a previous muscle biopsy, and the patient had been diagnosed with a frame-shift deletion of dystrophin exon 7 by multiplex ligation-dependent probe amplification (MLPA) analysis. The deletion of exon 7 leads to a premature translation termination at exon 8. The deletion of exon 9 is known as a common splice variant maintaining the reading frame in dogs and humans [17], [18] (**Figure 1A**). RT-PCR analysis of dystrophin mRNA using the patient's lymphocytes showed an exon 7 deletion, and direct sequence analysis of the RT-PCR products revealed a conjunction of exons 6 and 8 (**Figure 1B**). To determine the intron length, we performed a deletion breakpoint analysis. The genomic PCR roughly narrowed the breakpoint window to 2.5 kb between introns 6 and 7, then primer walking sequence analysis revealed the 50.4 kb deletion (Vega v35 chromosome X 32771568 to 32821979) [13] and the breakpoint accompanying an insertion of 13 bases of unknown origin (**Figure 1C**).

### Myogenic conversion of fibroblasts by MyoD transduction and selection of appropriate cell lineage for exon skipping

**Figure 2:**
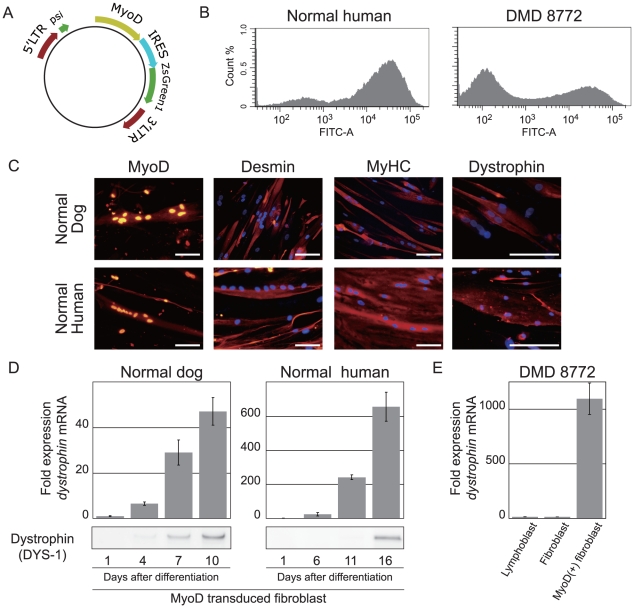
Myogenic conversion of fibroblasts and dystrophin expression. (A) Schematic diagram of the retroviral expression vector. (B) Histograms showing GFP fluorescence intensity compared with cell numbers of normal human and DMD 8772 MyoD-GFP-transduced fibroblasts. Both cells were analyzed five days after retroviral transfection. (C) Immunostaining of MyoD-transduced of dog and human fibroblasts after 10 and 15 days of myogenic differentiation, respectively. MyHC, myosin heavy chain. The nuclei were counter-stained with DAPI. Scale bar: 100 µm. (D) The time course of dystrophin expression in dog and human MyoD-transduced fibroblasts by qRT-PCR and immunoblotting analysis. The mRNA levels were normalized to *GAPDH* and expressed relative to the amount of the lowest one in each group. For immunoblotting, 5 µg of total protein was loaded into each lane. Error bars indicate standard deviation. (E) Determination of dystrophin mRNA expression in each cell type from DMD 8772 by qRT-PCR. MyoD-transduced fibroblasts were assayed 15 days after differentiation. Normalization and relative expression are the same as (D).

We prepared lymphoblastoid cells, fibroblasts, and MyoD-transduced fibroblasts from DMD 8772 and assessed the feasibility of exon skipping in these cells. To establish MyoD-transduced fibroblasts, primary fibroblasts were transfected by a retrovirus encoding murine or human MyoD and GFP with the vesicular stomatitis virus (VSV-G) envelope through standard procedures (**Figure 2A**) [19], [20]. To compare exon skipping between corresponding cells of CXMD_J_ and DMD 8772, fibroblasts from both were converted. In addition, normal dog and human fibroblasts were also transduced for evaluation. After virus transfection, we sorted GFP-positive cells by flow cytometry. The ratio of GFP-positive to -negative cells was dependent on cell lineage, and affected cells generally showed lower transfection efficiencies (**Figure 2B**). The GFP-positive cells were isolated in serum-deprived medium for myogenic differentiation and cultured for 10 to 16 days. We confirmed that the cultured cells had the morphological features of myotubes of multiple nuclei and longitudinal growth. Immunostaining analysis showed nuclear localization of MyoD and expressions of the muscle-specific proteins desmin, myosin heavy chain, and dystrophin (**Figure 2C**). Using normal dog and human fibroblasts, we performed time-course expression analyses of dystrophin mRNA by qRT-PCR and dystrophin protein by Western blot. The results showed a gradual increase in dystrophin expression. In dog cells, dystrophin became detectable on the protein level seven days after differentiation, whereas human cells required two weeks or more (**Figure 2D**). We compared the dystrophin mRNA expression of the lymphoblastoid cells, fibroblasts, and MyoD-transduced fibroblasts from DMD 8772. The MyoD-transduced fibroblasts showed remarkable expression compared with the other cells (**Figure 2E**). We tried exon skipping in lymphoblastoid cells, fibroblasts and MyoD-transduced fibroblasts, but only the MyoD-transduced fibroblasts yielded reproducible results. The lymphoblastoid cells and fibroblasts often failed to produce PCR products, and the skipped in-frame products were undetectable even if PCR products were generated (**data not shown**). Therefore, we used MyoD-transduced fibroblasts in the subsequent assays.

### Antisense PMO sequence design

**Figure 3:**
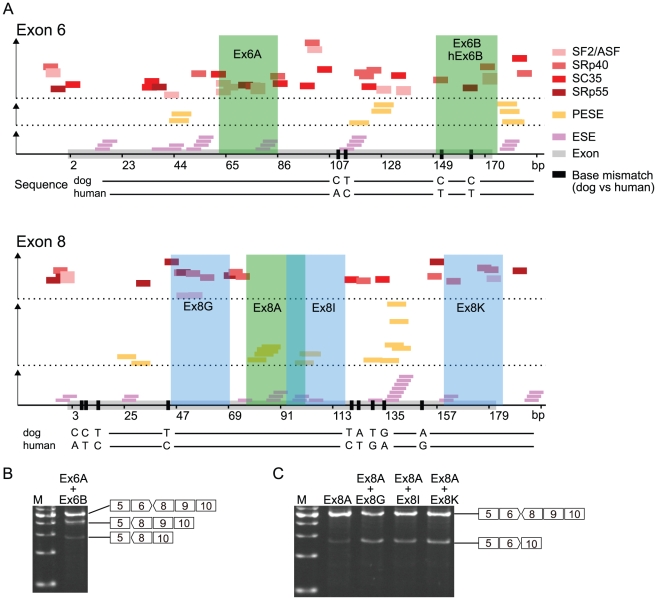
Design of antisense PMO sequence targeting exons 6 and 8. (A) Exonic splicing enhancer motifs predicted *in silico* based on human sequence (small coloured boxes) and positions of antisense PMOs (green and blue rectangular areas). The horizontal axis represents base positions in each exon from 5′ to 3′, and the vertical axis represents relative predicted values of the motifs. PESE: putative exonic splicing enhancer. ESE: exonic splicing enhancer. Base mismatches between dog and human (black bar) are indicated in the exon (grey box). RT-PCR of dystrophin mRNA of MyoD-transduced CXMD_J_ fibroblasts treated with (B) a mixture of Ex6A and Ex6B and (C) only Ex8A or mixtures containing Ex8A.

In a previous systemic dog study, we used three antisense sequences, Ex6A, Ex6B, and Ex8A, as three antisense PMO cocktails [6]. Because there were two base mismatches between dog and human Ex6B, hEx6B was newly designed on the identical region of Ex6B, modifying the mismatches of the human sequence. In the systemic study, we skipped exon 6 with a combination of Ex6A and Ex6B, and thus we tried same strategy for exon 8. We newly designed several antisense PMOs targeting exon 8 that were positioned on the identical sequence in dog and human considering the predicted *in silico* splice-enhancer motifs (**Figure 3A**). A preliminary assay of CXMD_J_ cells showed that three sequences, Ex8G, Ex8I, and Ex8K, were effective. Therefore, the antisense combination for exon 8 contained an extra antisense sequence from Ex8G, Ex8I, or Ex8K in addition to that of Ex8A. The skipping efficacy of each combination was higher than that of Ex8A alone, and those of Ex8G, Ex8I, and Ex8K were comparable (**Figure 3C**).

### Comparison of multiple skipping of exons 6 and 8 between CXMD_J_ and DMD 8772 cells

**Figure 4:**
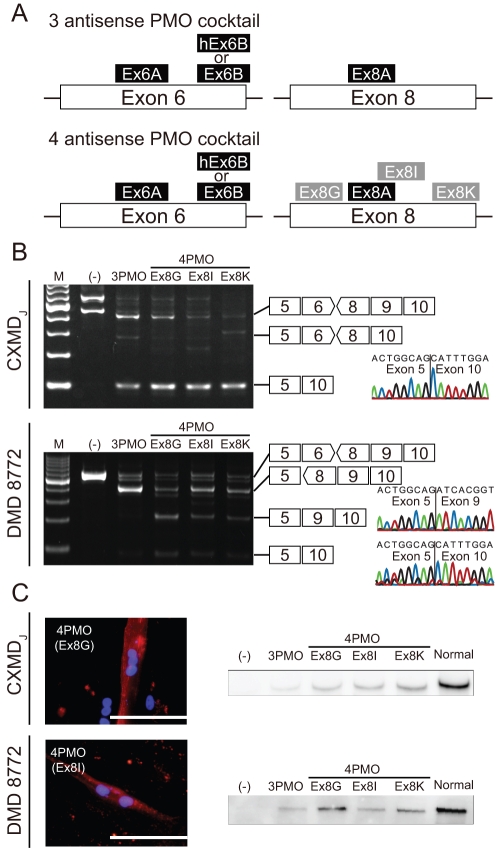
Multi exon skipping and recovery of dystrophin in CXMD_J_ and DMD 8772-derived cells. (A) Schematic diagram of the three- and four-antisense PMO cocktails. For DMD 8772, Ex6B was replaced with hEx6B. In the four-antisense PMO cocktail, one additional sequence (Ex8G, Ex8I, or Ex8K) was added to the three-antisense PMO cocktail. (B) RT-PCR of dystrophin mRNA isolated from MyoD-transduced fibroblasts after treatment with the three- and four-antisense PMO cocktails. In-frame exon skipping products were 99 bp in dog and 221 bp and 92 bp in human. (C) Representative immunostaining and immunoblotting analysis of MyoD-transduced fibroblasts treated with antisense PMO cocktails. The nuclei were counterstained with DAPI. Scale bar: 100 µm. Expected molecular weights of truncated human dystrophin with exons 6–8 and exons 6–9 skipped are 18.3 kDa and 23.1 kDa, respectively, smaller than the full-length dystrophin.

The multi-exon skipping of exons 6 and 8 employed three- and four-antisense PMO cocktails. In the three-antisense PMO cocktail for dogs, Ex6A, Ex6B, and Ex8A were included, and Ex6B was replaced with hEx6B for the human. The four-antisense PMO cocktail included one of Ex8G, Ex8I, or Ex8K in addition to the three-antisense PMO cocktail (**Figure 4A**). When we transfected the three- or four-antisense PMO cocktails into the MyoD-transduced fibroblasts, we did not observe the skipped products (231 bp) of exons 6-8 on RT-PCR analyses of CXMD_J_ but did observe the skipped products (99 bp) of exons 6-9. A sequence analysis also confirmed the concatenation of exons 5 and 10. In DMD 8772, we observed skipped products (221 bp and 92 bp, respectively) of both exons 6-8 and exons 6-9. Sequence analysis also showed that the skipped products were concatenations of exons 5 to 9 and exons 5 to 10. The four-PMO cocktails produced more in-frame products than the three-PMO cocktail, but we discerned no difference among the four PMO cocktails. This tendency was also consistent between CXMD_J_ and DMD 8772 (**Figure 4B**). Immunostaining analysis showed partial recovery of dystrophin in the four-antisense PMO cocktail-treated cells without obvious differences between them (**Figure 4C**). Western blots of dystrophin showed products that were slightly smaller than the full-length dystrophin. In RT-PCR of DMD 8772, skipped mRNA of both exons 6-8 and 6-9 were detected; however, distinguishing the truncated dystrophins translated from these mRNA variants was impossible. Similar to the RT-PCR results, the dystrophin expression level was higher with a four-PMO cocktail than with the three-PMO cocktail. Differences between the four-PMO cocktails were also undetectable.

## Discussion

In this study, we accomplished *in vitro* multi-exon skipping in a DMD patient carrying the same deletion as CXMD_J_ by using the identical antisense PMO. We also addressed the efficient MyoD transduction of fibroblasts with FACS, and discuss the difference of the spliced exon associated with it with the frequency of alternative splicing.

### FACS-aided MyoD transduction provided sufficient dystrophin expression

We evaluated the appropriateness of lymphoblastoid cells, fibroblasts, and MyoD-transduced fibroblasts as an alternative to myoblasts for exon-skipping assays. Lymphoblastoid cells and primary fibroblasts dystrophin mRNA required reamplification by nested RT-PCR [9], [10], and the results were not reproducible, suggesting that low dystrophin expression may hamper reliable quantitative assessments. Only MyoD-transduced fibroblasts showed reproducible results due to their stable dystrophin expression. We employed flow cytometry for selection of MyoD-positive cells; it seems to offer several advantages against conventional drug-resistance selection. First, the transfection ratio in drug-resistance selection remains unknown until a selective drug is added. In contrast, with MyoD-transduced fibroblasts, we were able to roughly determine the ratio by fluorescence microscopy and adjust the culture scale to meet the size of the assay. Second, a low rate of myotubes formation after drug-resistance selection has been reported [21]. Our method actively selects MyoD-positive cells and enables pure clusters of MyoD-positive cells to form myotubes efficiently. MyoD transduction with GFP has been reported in several studies [22], [23] but not in dystrophin exon-skipping studies. We demonstrated that it is a suitable approach for the exon-skipping assay here as well. Several studies have reported difficulties inducing dystrophin in human cells with MyoD transduction [14], [15], [16]. In our experience, the typical morphological features of myotubes, multiple nuclei and longitudinal cell growth, do not necessarily indicate sufficient dystrophin expression. Seeding MyoD-positive cells at high density (>5.0×10^4^ cells/cm^2^) and incubating for longer periods (>2 weeks) were critical to induce sufficient dystrophin expression. Detachment of differentiated myotubes from culture wells was also problematic; supporting them with a coating matrix seems to promise better results.

### Direct translation of antisense PMO from dog to human was feasible

We previously reported systemic multi-exon skipping in CXMD_J_ with a 3-antisense PMO cocktail and amelioration of dystrophic pathology [6]. The effectiveness of the 3-antisense PMO cocktail was confirmed in MyoD-transduced fibroblasts derived from DMD 8772 as well. When the dog and human sequences were compared, 97% of dystrophin exon 6 and 95% of dystrophin exon 8 matched on the sequence level. This similarity enabled use of the unified antisense design methodology targeting the same sequence. We demonstrated that the identical antisense PMO sequence designed for dog and achieved multi-skipping of exons 6 and 8 in human cells. The skipping efficacies of the PMOs were indistinguishable between CXMD_J_ and DMD 8772; the superior efficacy of the four-PMO cocktail against that of the three-PMO cocktail and the equivalent efficacies of each four-PMO cocktail were comparable. CXMD_J_ shows more similarity in the pathogenic phenotype to human DMD than to *mdx* mice [24]. These findings imply that not only the similarity in the sequence but also the similarity in the pathogenic phenotype contributed to the comparable results.

No study has yet compared the exon skipping due to identical antisense PMOs between cells of different species carrying same exon deletion in mRNA. Recent investigations have reported a limitation in designing efficient antisenses to induce human dystrophin skipping in a mice model assay [25]; however, we confirmed the feasibility of direct translation of an antisense PMO from a DMD dog model to a DMD patient, at least *in vitro*, for the first time.

The four-antisense PMO cocktail, the addition of a fourth antisense sequence to the three-antisense PMO cocktail, increased the efficiency of skipping as previously reported [26], [27]. The effectiveness of the four-antisense PMO cocktails, however, must be evaluated *in vivo*, and we are planning systemic treatment of CXMD_J_ with them. Our results underscore the usefulness of CXMD_J_ as a DMD model for translational research and advance the prospect that systemic treatment of the DMD patient by multi-exon skipping is possible.

### Mode of exon 9 skipping might be affected by frequency of alternative splicing

**Figure 5:**
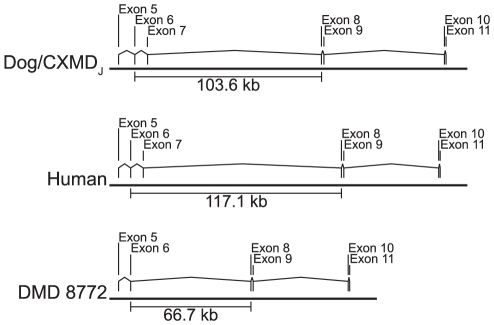
Location of dystrophin exons 5 to 11 in the genome. Distances from dystrophin exon 6 to exon 8 are indicated based on the GenBank reference sequences of *Canis familiaris* chromosome X genomic contig, whole genome shotgun sequence (NW_879562.1) and *Homo sapiens* 211000035840903 genomic scaffold, whole genome shotgun sequence (CH471074.1).

With the antisense PMO targeting exons 6 and 8, exon 9 was always skipped in CXMD_J_, although it was only partially skipped in DMD 8772. Two possibilities were considered to explain the difference: (1) the effects of the shortened introns 6 and 7 due to the deletion around exon 7 in DMD 8772 (**Figure 5**), and (2) the different frequencies of alternative splicing of exon 9. For the former case, we tried exon 8 skipping using a combination of Ex8A and Ex8G in normal and affected human MyoD-transduced fibroblasts, and found that the skipping of exon 8 and exons 8/9 happened simultaneously (**Figure S1**). Therefore, it is unlikely that the intron length affects the difference. In the latter case, the untreated MyoD-transduced fibroblasts from CXMD_J_ clearly showed one normal and one alternative transcript; on the other hand, the untreated sample from DMD 8772 showed only a normal transcript, suggesting that the frequency of alternative splicing of exon 9 is an underlying factor in the difference. It was reported that an antisense oligonucleotide targeting exon 8 facilitates the skipping of exon 9 as well as exon 8 by effecting the concatenation of exons 8 and 9 in human and dog cells [28]. These findings were observed in myoblasts but not in MyoD-tansduced fibroblasts [29], [30], [31]. As is well known, the mode of alternative splicing differs among various tissues [32], [33], and our MyoD-transduced fibroblasts might have characteristics that are incompatible with the alternative splicing of exon 9.

In summary, MyoD transduction of fibroblasts with the help of FACS may be practical for exon skipping assays, and the direct translation of an antisense PMO from a DMD dog model to a DMD patient was feasible *in vitro*, suggesting that the animal model-based antisense PMO for multiple skipping could be effective for humans as well.

## Materials and Methods

### Ethics Statement

The patient samples were collected and used with the approval of the Ethics Committee of the National Center of Neurology and Psychiatry, approval ID: 20-4-6. Written informed consent was obtained from the donor. The dog study was approved by the Ethics Committee for the Treatment of Middle-sized Laboratory Animals of the National Center of Neurology and Psychiatry, approval ID: 20-05.

### Cell culture

Dog primary myoblasts and fibroblasts were obtained from muscle specimens of normal and affected neonatal dogs of the CXMD_J_ colony using a standard pre-plating technique. Primary fibroblasts of the DMD patient (DMD 8772) were obtained from skin explants and peripheral blood lymphocytes using Lymphocyte Separating Medium (PAN Biotech GmbH, Aidenbach, Germany). Lymphoblastoid cell lines were established by transformation with Epstein-Barr virus. The normal human fibroblast cell line TIG-119 was obtained from the Health Science Research Resource Bank (Osaka, Japan). Fibroblasts were cultured in 20% or 10% growth medium containing DMEM/F-12 1∶1 (Invitrogen, San Diego, CA, USA), 20% or 10% fetal bovine serum, and 1% penicillin/streptomycin. For differentiation to myotubes, FACS-sorted MyoD-transduced fibroblasts were cultured in 2% differentiation medium containing DMEM/F-12 1∶1, 2% horse serum, ITS Liquid Media Supplement (Sigma-Aldrich, St. Louis, MO, USA), and 1% penicillin/streptomycin.

### Genomic mutation analysis

The dystrophin exon 7-deletion of DMD 8772 had been identified previously by MLPA. For breakpoint detection, lymphocyte genomic DNA was used as a template. Seven pairs of intron-spanning primers, positioned in the intron 6/7, were designed to yield 150-600 bp PCR products. A failure of PCR indicated deletions spanning the primer annealing sites. Four of seven primer pairs showed no amplification, suggesting that the deletion was more than 3.5 kb and less than 64.4 kb. Additionally, two intron 6 sense-primers and eight intron 7 antisense-primers were designed. Each primer pair was placed by flanking the breakpoint and expected to yield PCR products within the range of 4–64 kb. Primer sequences are available on request. PCR was performed using Phusion Hot Start High-Fidelity DNA Polymerase (Finnzymes, Keilaranta, Finland), and the cycling program was set to yield 16 kb products with a program of 35 cycles of 98°C for 10 sec, 60°C for 30 sec, and 72°C for 450 sec. Failure of PCR indicated products of more than 16 kb in size or the deletion of annealing sites. The breakpoint region was thus narrowed down to 2.5 kb, then primer walk sequencing was performed (Operon Biotechnologies, Tokyo, Japan).

### MyoD transduction and cell sorting by FACS

The coding sequences of mouse *Myod1* (CCDS 21277.1) and human *MYOD1* (CCDS 7826.1) were derived from the Consensus CDS database [34]. The sequences were synthesized and cloned into a pUC57 vector (GenScript, Piscataway, NJ, USA). We subcloned it into a pRetroX-IRES-ZsGreen1 expression vector (Clontech, Mountain View, CA, USA). The expression vector, a pVSV-G envelope vector, and a gap-pol expression vector were co-transfected into a 293T packaging cell line using the standard calcium phosphate method. After 48–72 h incubation, the viral supernatant was collected and stored at −80°C. For retroviral transduction, the fibroblasts were harvested at 70–80% confluence in a T225 flask, and 2.5 ml thawed retroviral stock was added to 35 ml of growth medium. We added polybrene (Sigma-Aldrich) to a final concentration of 8 µg/ml. After 48–72 h incubation at 32°C, the culture medium was replaced with fresh growth medium, the cells were incubated at 37°C 1–3 d more, until the GFP-positive cells exceeded approximately 60%. Cell sorting was performed on a FACS VantageSE or FACSAria flow cytometry system (BD Bioscience, Franklin Lakes, NJ, USA). The recovered GFP-positive cells were seeded in Matrigel (BD Bioscience)-coated well plates at density of 5×10^4^ cell/cm^2^. After confirmation of cell attachment, the culture medium was changed to 2% differentiation medium. We cultured MyoD-transduced fibroblasts for 10 to 16 d to differentiate to myotubes.

### Antisense PMO design and transfection to cultured cells

The antisense PMO sequences Ex6A, Ex6B, and Ex8A were described in Yokota et al. [6]. In addition, extra sequences hEx6B, Ex8G, Ex8I, and Ex8K were designed and synthesized (Gene Tools, LLC, Philomath, OR, USA). We used the Human Splicing Finder for *in silico* prediction of the splice-enhancer motifs [35]. All sequences are shown in **Table S1**. We transfected the antisense PMOs into myotubes differentiated from MyoD-transduced fibroblasts with a transfection agent, Endo-Porter (Gene Tools). In the 2% differentiation medium, the final concentration of the antisense PMO was 10 µM for a single sequence, 20 µM for two sequences, and a total of 30 µM for three or four sequences. A final concentration of Endo-Porter was 6 µM. After 48–72 h incubation with the PMO, the medium was changed to a fresh culture medium free of PMOs. The cells were recovered for analysis after 24–48 h in the PMO-deprived medium to allow sufficient time to translate dystrophin protein.

### Quantitative RT-PCR analysis

Total RNA was extracted from MyoD-transduced fibroblasts obtained from normal subjects using Trizol (Invitrogen) at the time points specified. Total RNA (100-200 ng) was employed for cDNA synthesis using a QuantiTect Reverse Transcription Kit (Qiagen, Hilden, Germany). Quantitative real-time PCR was performed using ExTaq II SYBR (Takara, Kyoto, Japan) and a MyiQ Single-Color Real-Time PCR detection system (Bio-Rad, Hercules, CA). Primer sequences are shown in **Table S2**. Expression of dystrophin mRNA was normalized to *GAPDH* mRNA, and the time course of the increment was calculated by the delta-delta-Ct method.

### RT-PCR and sequence analysis

As well as quantitative RT-PCR analysis, total RNA extraction and cDNA synthesis were performed. For myoblasts and MyoD-transduced fibroblasts, 35 cycles of denaturing at 98°C for 10 sec, annealing at 63°C for 30 sec, and extension at 72°C for 1 min were performed with ExTaq DNA polymerase (Takara). For fibroblasts and lymphoblasts, nested PCR was performed. Primer sequences are shown in **Table S3**. PCR products were electrophoresed on 1.2% SeaKem LE agarose gel (Lonza, Basel, Switzerland). The bands of interests were excised using a Wizard SV Gel and PCR Clean-Up system (Promega, Fitchburg, WI, USA), then sequenced directly or cloned into a vector using a TOPO-TA Cloning Kit (Invitrogen) with standard cloning techniques. Sequencing was performed by Fasmac Corporation (Kanagawa, Japan).

### Immunostaining analysis

Cells were fixed in 3% paraformaldehyde, permeabilized in 10% Triton-X, then blocked by 10% goat serum in PBS for 1 h at room temperature. The cells were incubated with the primary antibody for 1 h at room temperature using anti-dystrophin (NCL-Dys1, diluted 1∶30, Novocastra, Newcastle upon Tyne, UK), anti-myosin heavy chain (NCL-MHCf, diluted 1∶30, Novocastra), anti-MyoD (NCL-MyoD1, diluted 1∶30, Novocastra), or anti-desmin (NCL-DES-DERII, diluted 1∶30, Novocastra). Incubation with the secondary antibody was performed for 30 min at room temperature using anti-rabbit or anti-mouse IgG (Alexa Fluor 546 highly cross-adsorbed, diluted 1∶300, Invitrogen). Antibodies were diluted in Can Get Signal Immunostain A solution (Toyobo, Osaka, Japan). To visualize nuclei and enhance fluorescence signals, cells were mounted with Pro Long Gold Antifade reagent (Invitrogen).

### Immunoblotting analysis

Protein was extracted from cultured cells using RIPA buffer (Thermo Fisher Scientific, Rockford, IL, USA) containing Complete Mini (Roche Applied Science, Indianapolis, IN, USA) as a protease inhibitor. Protein concentrations were determined using a BCA protein assay kit (Thermo Fisher Scientific) and equalized. After being mixed with an equal volume of EzApply sample buffer (ATTO Corporation, Tokyo, Japan), cell lysates containing equal amounts of total protein were denatured at 95°C for 5 min, electrophoresed in NuPAGE Novex Tris-Acetate Gel 3–8% (Invitrogen) at 150 V for 75 min, and transferred onto an Immobilon-P membrane (Millipore Corp., Billerica, MA, USA). Membranes were blocked for 1 h with 5% ECL Blocking agent (GE Healthcare, Buckinghamshire, UK) and probed with anti-dystrophin antibody (NCL-Dys1, diluted 1∶50, Novocastra), followed by incubation with peroxidase-conjugated goat-anti-mouse IgG (Bio-Rad). An ECL Plus Western blotting system (GE Healthcare) was used to detect protein bands.

## Supporting Information

Figure S1 RT-PCR of dystrophin mRNA isolated from the normal and affected human MyoD-transduced fibroblasts after the single exon 8 skipping.
(0.30 MB PDF)

Table S1 Sequences of antisense PMO for dystrophin gene (for dog and human if not specified).
(0.07 MB PDF)

Table S2 Sequences of qRT-PCR primers.
(0.07 MB PDF)

Table S3 Sequences of RT-PCR primers.
(0.07 MB PDF)
