# Supplementary material for: Antisense PMO Found in Dystrophic Dog Model Was Effective in Cells from Exon 7-Deleted DMD Patient
Source: PLoS One. 2010 Aug 18;5(8):e12239. doi: 10.1371/journal.pone.0012239 (PMC2923599; doi:10.1371/journal.pone.0012239)
Supplement: Figure S1 — RT-PCR of dystrophin mRNA isolated from the normal and affected human MyoD-transduced fibroblasts after the single exon 8 skipping. (0.30 MB PDF) [file pone.0012239.s001.pdf]

Supporting information

Supplemental Figure S1. RT-PCR of dystrophin mRNA isolated from normal and affected human MyoD-transduced fibroblasts after single exon 8 skipping.

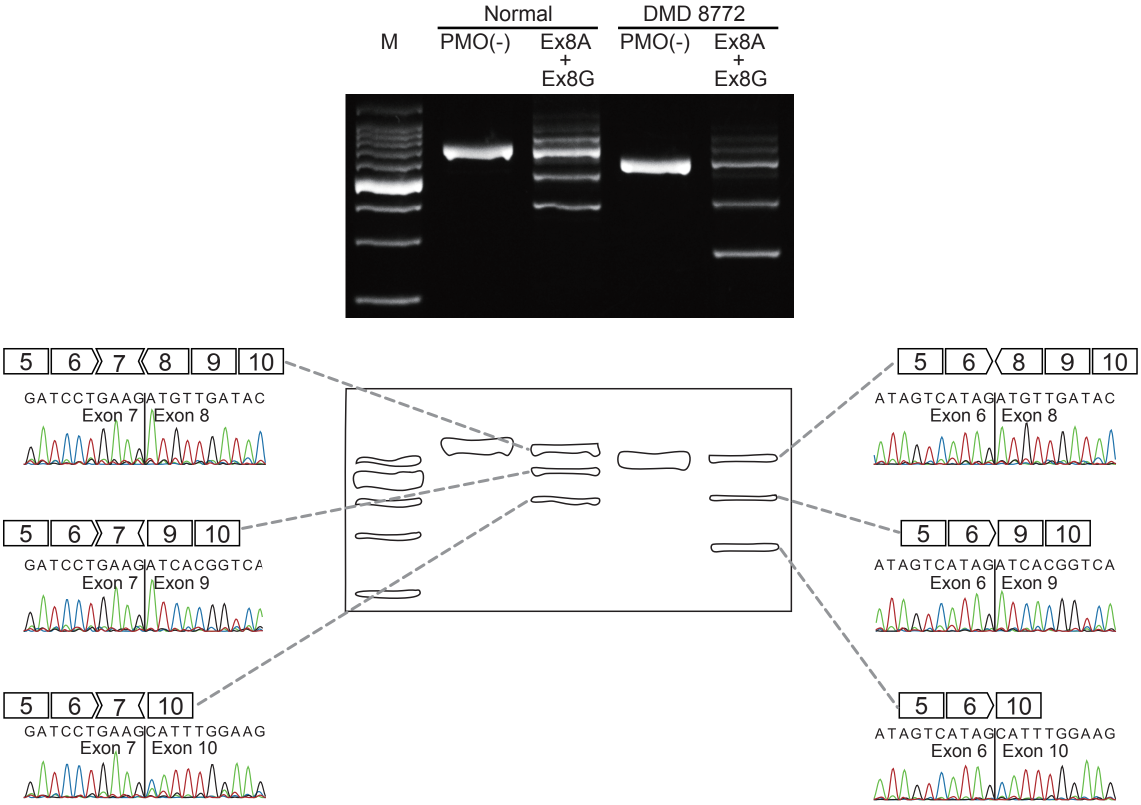

Cells were treated with the combination of Ex8A and Ex8G in a final concentration of total 20 µM. PCR products sizes were 695 bp (no skip), 513 bp (exon 8 skip), and 384 bp (exons 8 and 9 skip) for the normal subject and 576 bp (exon 7 skip), 394 bp (exons 7 and 8 skip), 265 bp (exons 7, 8 and 9 skip) for DMD 8772. All product sequences are shown.
