# Supplementary material for: Antisense PMO Found in Dystrophic Dog Model Was Effective in Cells from Exon 7-Deleted DMD Patient
Source: PLoS One. 2010 Aug 18;5(8):e12239. doi: 10.1371/journal.pone.0012239 (PMC2923599; doi:10.1371/journal.pone.0012239)
Supplement: Table S1 — Sequences of antisense PMO for dystrophin gene (for dog and human if not specified). (0.07 MB PDF) [file pone.0012239.s002.pdf]

Supporting information

Supplemental Table S1. Sequences of antisense PMO for dystrophin gene (for dog and human if not specified).

Ex6A	GTTGATTGTCGGACCCAGCTCAGG
Ex6B (for dog)	ACCTATGACTGTGGATGAGAGCGTT
Ex8A	CTTCCTGGATGGCTTCAATGCTCAC
Ex8G	GGCAAAACTTGGAAGAGTGATGTGA
Ex8I	CCTTGGCAACATTTCCACTTCCTGG
Ex8K	TTTACCTGTTGAGAATAGTGCATTT
hEx6B (for human)	ACCTATGACTATGGATGAGAGCATT
