# Supplementary material for: Antisense PMO Found in Dystrophic Dog Model Was Effective in Cells from Exon 7-Deleted DMD Patient
Source: PLoS One. 2010 Aug 18;5(8):e12239. doi: 10.1371/journal.pone.0012239 (PMC2923599; doi:10.1371/journal.pone.0012239)
Supplement: Table S2 — Sequences of qRT-PCR primers. (0.07 MB PDF) [file pone.0012239.s003.pdf]

Supporting information

Supplemental Table S2. Sequences of qRT-PCR primers.

Primers for dog dystrophin qRT-PCR	
d*DMD*2/3jnc Fwd	AAGTTTGGGAAGCAGCACAT
d*DMD*4 Rev	GGGCATGAACTCTTGTGGAT
d*GAPDH* Fwd	GAGTCCACTGGGGTCTTCA
d*GAPDH* Rev	GAGGAGCCAAGCAGTTGGT
Primers for human dystrophin qRT-PCR	
h*DMD*3 Fwd	TGGGAAGCAGCATATTGAGA
h*DMD*4 Rev	GGGCATGAACTCTTGTGGAT
h*GAPDH* Fwd	GAGTCCACTGGCGTCTTCA
h*GAPDH* Rev	GGGGTGCTAAGCAGTTGGT
