# Supplementary material for: Antisense PMO Found in Dystrophic Dog Model Was Effective in Cells from Exon 7-Deleted DMD Patient
Source: PLoS One. 2010 Aug 18;5(8):e12239. doi: 10.1371/journal.pone.0012239 (PMC2923599; doi:10.1371/journal.pone.0012239)
Supplement: Table S3 — Sequences of RT-PCR primers. (0.07 MB PDF) [file pone.0012239.s004.pdf]

Supporting information

Supplemental Table S3. Sequences of RT-PCR primers.

Primers for dog dystrophin exons 5-10 RT-PCR	
dh*DMD*5 Fwd	CTGACTCTTGGTTTGATTTGGA
d*DMD*10 Rev	TGCTTCGGTCTCTGTCAATG
Primers for human dystrophin RT-PCR	
Amplification of exons 5-10	
dh*DMD*5 Fwd	CTGACTCTTGGTTTGATTTGGA
h*DMD*10 Rev	CTCTCCATCAATGAACTGCC
Pre-amplification of exons 4-11 for nested RT-PCR	
h*DMD*4 Fwd	ATCCACAAGAGTTCATGCCC
h*DMD*11 Rev	ATGCTAGCTACCCTGAGGCA
